# Potential role of probiotics on colorectal cancer prevention

**DOI:** 10.1186/1471-2482-12-S1-S35

**Published:** 2012-11-15

**Authors:** Mario Uccello, Giulia Malaguarnera, Francesco Basile, Velia D’agata, Michele Malaguarnera, Gaetano Bertino, Marco Vacante, Filippo Drago, Antonio Biondi

**Affiliations:** 1 Department of Senescence, Urological and Neurological Sciences, Cannizzaro Hospital Via Messina 829, 95125, University of Catania, Italy; 2 International PhD programme in Neuropharmacology, University of Catania, Italy; 3 Department of General Surgery, Section of General Surgery and Oncology, Vittorio Emanuele Hospital, Via Plebiscito 628 University of Catania, 95123 Catania, Italy; 4 Department of Biomedical Sciences, Via S. Sofia, 87, 95123, University of Catania, Italy. University of Catania, Italy; 5 Department of Medical and Pediatric Sciences Via S. Sofia, 87, 95123, University of Catania, Italy

## Abstract

**Background:**

Colorectal cancer represents the most common malignancy of the gastrointestinal tract. Owing to differences in dietary habits and lifestyle, this neoplasm is more common in industrialized countries than in developing ones. Evidence from a wide range of sources supports the assumption that the link between diet and colorectal cancer may be due to an imbalance of the intestinal microflora.

**Discussion:**

Probiotic bacteria are live microorganisms that, when administered in adequate amounts, confer a healthy benefit on the host, and they have been investigated for their protective anti-tumor effects. In vivo and molecular studies have displayed encouraging findings that support a role of probiotics in colorectal cancer prevention.

**Summary:**

Several mechanisms could explain the preventive action of probiotics against colorectal cancer onset. They include: alteration of the intestinal microflora; inactivation of cancerogenic compounds; competition with putrefactive and pathogenic microbiota; improvement of the host’s immune response; anti-proliferative effects via regulation of apoptosis and cell differentiation; fermentation of undigested food; inhibition of tyrosine kinase signaling pathways.

## Background

Colorectal cancer [CRC] is one of the major health problems in the world, representing the most common malignancy of the gastrointestinal [GI] tract. CRC is more frequent in industrialized countries than in developing ones with a four times higher incidence [1]. Differences in dietary habits and lifestyle rather than racial factors may explain this gap as it has been demonstrated by studies on migrants. The diet is likely to play a key role in the pathogenesis of CRC. Epidemiological studies have shown that the consumption of red meat and animal fat is associated with an increased risk for CRC development [2], whereas a diet rich in fruits and vegetables appears to be protective against CRC [3]. Evidence from a wide range of sources supports the assumption that the link between diet and CRC may be due to an imbalance of the intestinal microflora [4]. At birth, the GI tract is colonized by microbes and remains the home for several populations of microorganisms throughout the life of the host. The ‘normal’ gut microflora consists of bacterial species with morphological, physiological and genetic features that let it to colonize and multiply under particular conditions at certain sites, coexist with other colonizing microorganisms and competitively inhibit the growth of pathogenic bacteria. Nevertheless, some environmental factors such as diet and drugs can alter the composition of the resident microbiota, with consequent dysmicrobia and negative implications for the health of the individual. The colonic microflora is very rich and dominated by strict anaerobic bacteria such as *Bacteroides spp.*, *Fusobacterium spp.*, *Clostridium spp*, and many others [5]. Probiotic bacteria may be defined as ‘live microorganisms which when administered in adequate amounts confer a health benefit on the host’ [6], and they most frequently belong to the lactic acid bacteria [LAB] category, such as *Lactobacillus spp.* and *Bifidobacterium spp.* LAB are widely available, for instance, in yogurts and other functional foods such as cheese, fermented and unfermented milks, juices, smoothies, cereal, nutrition bars and infant/toddler formula [7]. A number of studies in animal models and in human population have demonstrated that the consumption of probiotics is effective in various medical conditions such as lactose intolerance, antibiotic-induced diarrhea, gastroenteritis, constipation, and genitourinary tract infections [8]. Moreover, accumulating evidence suggests that the ingestion of probiotics may be able to play a preventive role in the onset of CRC [4]. This observation seems to be very interesting as it would make possible an effective strategy for CRC primary prevention. This review is merely intended at providing an outline of the possible mechanisms whereby probiotics may exert their beneficial effects for CRC prevention. We have given greater emphasis on those novel mechanisms, such as the inhibition of tyrosine kinase signaling pathways and anti-proliferative effects, that have not been thoroughly discussed yet.

## Discussion

### Mechanisms of CRC prevention exerted by probiotics

Despite the great number of studies in the literature, the precise mechanisms by which probiotics may prevent CRC still remain not perfectly clear. However, it is conceivable that they include: alteration of the intestinal microflora; inactivation of cancerogenic compounds; competition with putrefactive and pathogenic microbiota; improvement of the host’s immune response; anti-proliferative effects via regulation of apoptosis and cell differentiation; fermentation of undigested food; inhibition of tyrosine kinase signaling pathways. The coadministration of probiotics with prebiotics [which are defined as ‘selectively fermented ingredients that allow specific changes, both in the composition and/or activity in the gastrointestinal microflora that confer benefits upon host well-being and health’ [9], the so-called synbiotics, can increase the effectiveness of these anti-cancer mechanisms [10,11]. Moreover, the acidification of pH, although not considered as a distinct mechanism of action, is an intrinsic and fundamental feature whereby many probiotics carry out their metabolic activities [12,13]. These potential mechanisms will be discussed individually now.

### Alteration of the intestinal microflora metabolism

Glucuronide conjugation is one of the major metabolic processes occurring in the liver. It is critical to metabolize hormones, and also to inactivate toxic and carcinogenic compounds of endogenous and exogenous origin. The conjugation with glucuronic acid results in polar metabolites that are efficiently eliminated in the bile [14]. The deconjugation of these glucuronides in the intestine by bacterial β-glucuronidase leads to the release of aglycones that are potentially carcinogenic substances [15]. There are other fecal bacterial enzymes, including azoreductase and nitroreductase, which catalyze the liberation of procarcinogenic substances in the intestine [16,17]. The alteration of the intestinal metabolism by modulating the activity of these bacterial enzymes may be one of the possible mechanisms by which probiotics may reduce the risk for the onset of CRC [18]. It has been demonstrated that a yogurt feeding can reduce the levels of β-glucuronidase and nitroreductase contained in the large intestine of mice bearing colon cancer [19]. Goldin and Gordbach [18] reported a decrease in fecal bacterial enzyme activity after a *Lactobacillus acidophilus* feeding in animal models. The same authors [20] recruited 21 young healthy subjects for a study aimed at investigating the effect of *L. acidophilus* oral supplements on the enzyme activity of β-glucuronidase, nitroreductase and azoreductase. Both two strains of *L. acidophilus* used in the study [N-2 and NCFM] caused a significant decrease in the activities of the three fecal enzymes after a ten-days lactobacilli feeding. Having stopped the bacterial feedings, fecal enzyme levels returned to normal after four weeks, suggesting that continuous ingestion of these organisms is required for these enzyme effects to be maintained in the microflora. However, apparently ambiguous or discordant results have been shown by most human studies designed to investigate the effects of probiotics supplementation on fecal enzyme bacterial activity [21-26]. For example, Marteau *et al. *[24] reported a decrease only in nitroreductase activity after a three weeks-period of ingesting a fermented dairy product containing *L. acidophilus*, *Bifidobacterium bifidum*, and mesophilic cultures [*Streptococcus lactis* and *Streptococcus cremoris*] while β-glucuronidase and azoreductase activities did not change. Indeed, these findings suggest that the capability of modulating fecal enzymes bacteria activity is a strain-specific characteristic for probiotics. The duration and amount of probiotic intake are other considerable factors. Moreover, the degree of relationship between the ability of probiotics to influence the bacterial metabolism and the prevention of CRC has to be better clarified.

### Inactivation of cancerogenic compounds

A meta-analysis of 15 prospective studies showed a relative risk of developing CRC of 1.28 for subjects with a higher consumption of red meat, when compared with people who eat red meat in lower quantities [2]. Several hypotheses have been proposed to explain this relationship. Heterocyclic aromatic amines [HCA], formed as a result of cooking meat at high temperatures, are among the substances called into question [27,28]. Intestinal microbiota can activate HCA to their active derivatives such as the mutagenic pyrolyzates 3-amino-1,4-dimethyl-5H-pyrido-[4,3-b]indole [Trp-P-1], 3-amino-1-methyl-5H-pyrido-[4,3-b]indole [Trp-P-2], 2-amino-3-methylimidazo[4,5-f]quinoline [IQ], 2-amino-1-methyl-6-phenylimidazo[4,5-b]pyridine [PhIP], 2-amino-3,4-dimethylimidazo[4,5-f]quinoline [MeIQ], and 2-amino-3,8-dimethylimidazo[4,5-f]quinoxaline [MeIQx] [29,30]. Such powerful mutagenic substances may act with the colonic mucosa, causing tumorigenic mutations [30]. LAB and other commensal bacteria have been found to bind or metabolize several carcinogens, including HCA and N-nitroso compounds. Binding and/or degradation well correlates with the reduction in mutagenicity observed after exposure of HCA to the bacterial strains [31-33]. According to the literature, the binding or degradation of HCA by probiotics could be one of the main mechanisms of removing carcinogens out of the human body. Orrhage *et al. *[32] studied the *in vitro* capacity of some LAB to bind mutagenic HCA formed during the cooking of protein-rich food. The binding of the mutagens Trp-P-2, PhIP, IQ and MeIQx by the bacterial strains was analyzed by HPLC. Trp-P-2 was almost completely and irreversibly bound while the binding of PhIP, a major mutagen in the western diet, reached about 50%. IQ and MeIQx were slightly less well bound. Sreekumar and Hosono [34,35] demonstrated that different strains of *Lactobacillus gasseri* and *Bifidobacterium longum* strongly bound Trp-P-1 and Trp-P-2. Oral supplementation with *L. acidophilus* NCFB1748 and *B. longum* BB536 decreased the bioavailability of Trp-P-2 in the GI tract and other several tissues in mice [36]. Cell fractions of *L. acidophilus* and *Bifidobacterium spp.* have been found to bind Trp-P-1 and decrease its genotoxicity [37]. Most data suggest that the binding of mutagens could be due to the bacterial cell wall [32,37,38] though the anti-mutagenic effect of *Lactobacillus plantarum* KLAB21 is mediated by three extracellular glycoproteins [39]. Challa *et al. *[40] demonstrated that a *B. longum* and lactulose feeding in rats significantly increased the activity of colonic glutathione S-transferase, which is one of the Phase II enzymes involved in the detoxification of toxic metabolites and carcinogens, and suppressed azoxymethane [AOM]-induced colonic aberrant crypt foci [ACF] that are preneoplastic markers. More recently, *Lactobacillus casei* DN 114001 has been shown to grow and survive in the presence of IQ, MelQx and PhIP and to decrease their concentrations [12].The probiotic ability to bind or metabolize toxic compounds depends on pH and other physicochemical conditions [12,32,33]. All these results indicate that the detoxification of cooked food mutagenic compounds, commonly found in the western meat-rich diet, may be one of the main mechanisms by which LAB antagonize the onset of CRC.

### Competition with putrefactive and pathogenic microbiota

The GI tract, particularly the colon, is very heavily populated with bacteria. Although most gut bacteria are benign, some species are pathogenic and may be involved in the onset of acute and chronic disorders, including CRC [41]. It is established that a diet rich in animal fat stimulates the growth of secondary bile salt-producing bacteria and further studies have shown that secondary bile salts are cytotoxic and carcinogenic [42,43]. A diet rich in red meat also facilitates the growth of sulfate-reducing bacteria producing hydrogen sulfide which experimentally is known to be genotoxic [44-46]. Putrefactive intestinal microbiota such as *Bacteroides spp*. and *Clostridium spp.* have been implicated in the pathogenesis of CRC [47] while numerous LAB have been shown to possess cancer-preventing attributes [31]. Rafter *et al. *[48] found that the synbiotic combination of a specific oligofructose-enriched inulin with probiotics on the fecal flora of polyp and colon cancer patients caused an increase in the number of some groups of LAB [*Bifidobacterium* in both groups and *Lactobacillus* in polyp patients], whereas the number of *Clostridium perfringens* in polyp patients significantly decreased. The consumption of probiotics alone have also proved effectiveness to cause changes in GI microflora, with a significant reduction of fecal putrefactive bacteria, such as coliforms, and an increase of LAB [49,50]. These effects may be mediated by adherence to enterocytes and the pH lowering [13,51]. Furthermore, O’Mahony *et al.* reported that the enteric flora modification in interleukin-10 [IL-10] knockout mice by probiotic *Lactobacillus salivarius* UCC118 resulted in a reduced prevalence of colon cancer [49]. Thus, probiotics may counteract CRC development also through a mechanism of competition with pathogenic intestinal microbiota.

### Improvement of the host’s immune response

The immune system plays an important role in the control of tumor promotion and progression. The close interaction of several elements of the immune system, such as antigen-presenting cells [APCs], and different subsets of T cells, B cells and natural killer [NK] cells, is critical for the generation of an effective anti-tumour immune response [52]. Besides other potential effects in the prevention of cancer, probiotics have been suggested to enhance the mucosal and system immune response [53]. In 1981, Yokokura [54] screened 26 strains of 14 different species of LAB for *in vivo* anti-tumor activities against a transplantable mouse sarcoma, and noticed that some of these strains had potent anti-tumor effects. Among them, especially *Lactobacillus casei* Shirota [LcS] showed a high potential. Since such strain is not directly cytotoxic to tumor cells *in vitro*, it has been postulated that its anti-tumor effects may be mediated by the enhancement of the host’s immune system [55]. This hypothesis has elicited further investigations on the anti-tumor and immunoregulatory action of LcS in various experimental models [56-58]. Oral administration of LcS has exhibited beneficial effects in both humans and animals as well as anti-tumor activity against human bladder cancer cells in clinical trials [59,60]. LcS has been shown to possess powerful anti-tumor and anti-metastatic effects on transplantable tumor cells and to suppress chemically-induced carcinogenesis in rodents. In particular, It has been noted that the intrapleural administration of LcS into tumor-bearing mice has induced the production of several cytokines, such as Interferon-γ [IFN-γ], interleukin-β [IL-1β] and tumor necrosis factor-α [TNF-α], leading to the inhibition of tumor growth and to an increased survival [58,61]. After LcS is ingested by the host, it is incorporated into M cells in Peyer’s patches and digested to form active components. In Peyer’s patches, macrophages or dendritic cells [DCs], after phagocytosing LcS, become able to produce several cytokines, especially TNF-α. Then, the components of LcS digested in Peyer’s patches are recognized through toll-like receptor 2 in APCs, and lead to the production of several cytokines that stimulate different responses in host immune cells [62]. Lcs has also exhibited a strong anti-tumor effect in mice by regulating the host immune response in a 3-methylcholanthrene [MC]-induced carcinogenesis model [63] that has been used to induce many tumors, including colon cancer model [64,65]. An LcS oral feeding of mice is likely to counteract MC-induced tumorigenesis by ameliorating the host immune responses which have been disrupted during MC carcinogenesis. A possible mechanism of carcinogenesis prevention is the proliferation and activation of NK cells [66]. NK cells are large granular lymphocytes derived from bone marrow, and have a critical role in immune surveillance against tumor development [67]. Other possible effector cells that may respond to LcS and other probiotics are DCs [62,68]: they represent important types of cells involved in the presentation of several antigens and in the production of cytokines [69]. In addition, oral administration of LcS has been shown to stimulate type 1 helper T cells, activate the cellular immune system, and inhibit the incidence of tumors and IgE production in mice [70]. More recently, it has been reported that LcS has suppressed murine tumorigenesis with potent elicitation to produce interleukin-12 [IL-12] by bone marrow-derived cells *in vitro *[71] and to inhibit of interleukin-6 [IL-6] production in the colonic mucosa [72]. In numerous studies, other probiotic strains have shown remarkable immunoprotective properties through the increase of specific and non-specific mechanisms that have anti-tumor effects. For instance, Lee *et al. *[73] reported that the administration for four weeks of *L. acidophilus* SNUL, *L. casei* YIT9029 and *B. longum* HY8001, for instance, increased the survival rate of mice injected with tumor cells. The increase of survival was correlated with an increase in cellular immunity as reflected by an augmentation in the number of total T cells, NK cells and MHC class II^+^ cells, and CD4^−^CD8^+^ T cells in flow cytometry analysis. These findings suggest that the treatment with probiotics has the potential to prevent CRC by modulation of the host's immune system, specifically cellular immune responses.

### Anti-proliferative effects via regulation of apoptosis and cell differentiation

Apoptosis is a genetically determined mode of cell death playing a key role in the regulation of cell numbers. In many types of cancer, a reduced ability to trigger apoptosis is an important pathogenetic event that is accompanied by alteration of control processes of cell proliferation [74]. The regulation of cell survival and death with molecules acting on the apoptotic process can have a huge chemopreventive and therapeutic potential [75]. There is much evidence that probiotics can have a role in the regulation of cell proliferation and apoptosis which are potentially crucial mechanisms in the prevention of CRC. Iyer *et al. *[76] found that *Lactobacillus reuteri* suppressed TNF-induced NF-κB activation in a dose and time-dependent manner. *L. reuteri* may regulate cell proliferation by promoting apoptosis of activated immune cells via inhibition of IkBa ubiquitination and enhancing pro-apoptotic mitogen activated protein kinase [MAPK] signaling. The probiotic mixture VSL#3 has been reported to suppress the COX-2 expression in Colo320 and SW480 intestinal epithelial cells [77]. The expression of COX-2 is increased in colorectal tumors [78], and this elevation can protect intestinal epithelial cells from apoptosis [79,80]. Recently, rodent studies have demonstrated that the synbiotic combination of resistant starch and *Bifidobacterium lactis* has exerted a pro-apoptotic action in response to the carcinogen, AOM [10,81]. Other studies have postulated that probiotics possess CRC-protective effects by altering the differentiation process of tumor cells. Using a cultured human colon cancer cell line [HT-29], Baricault *et al. *[82] studied the effect of fermented milks on colon cancer cell proliferation and growth. Milks were fermented by one of the following bacterial populations: *Lactobacillus helveticus*, *Bifidobacterium*, *L. acidophilus* or a mix of *Streptococcus thermophilus* and *Lactobacillus bulgaricus*. After HT-29 cells were added to the fermented milk, only *L. acidophilus* was found to have no effects on both cell growth and differentiation while the three other bacterial strains induced a significant, although variable, reduction in the growth rate of HT-29 cells, which resulted in a 10-50% decrease in the cell number at steady-state. Concomitantly, the specific activities of dipeptidyl peptidase IV, which is a sensitive and specific marker of HT-29 cell differentiation, and those of three other brush border enzymes [sucrase, aminopeptidase N and alkaline phosphatase] were significantly increased, thus suggesting that these cells may have entered a differentiation process. Moreover, the combination of *Bifidobacterium breve* R0070 + *Lactobacillus lactis* R1058 + oligoalternan inhibited the proliferation of HT-29 cells in absence of cytotoxic effect [83]. This could be explained by the induction from an undifferentiated phenotype to a more differentiated one. In fact, the results showed that cancerous HT-29 cells treated with the synbiotic, when compared with the differentiated ones, reached the same rate expression of intestinal alkaline phosphatase, a biomarker of colic differentiation [84]. Singh *et al. *[85] demonstrated that a dietary administration in rats of lyophilized cultures of *B. longum* resulted in a significant suppression of colon tumor incidence and tumor multiplicity, and it also reduced the tumor volume. Analyses on intermediate biomarkers also revealed that the ingestion of *B. longum* inhibited AOM-induced cell proliferation through a reduction in ornithine decarboxylase [ODC] activity. ODC is involved in the biosynthesis of polyamines that cause cell proliferation and differentiation of the colonic mucosa [86]. According to these data, an improved understanding of LAB-mediated effects on apoptosis and differentiation signalling pathways may facilitate the development of future probiotics-based regimens for the prevention of CRC.

### Fermentation of undigested food

The bacterial transformation of dietary components in the intestinal lumen may be associated with the production of cancer-preventive agents and may therefore be another mechanism whereby probiotics can influence CRC risk. The bacterial fermentation of indigestible carbohydrates generates short-chain fatty acids [SCFA] and gas; while the gas is eliminated in the feces, SCFA [mainly acetate, propionate and butyrate] represent nutrients and growth signals for the intestinal mucosa and may play a role in CRC prevention [87]. They reduce, for instance, the concentration of secondary bile salts. Butyrate, that is the most widely studied of these SCFA, is a preferred energy source for colonocytes and is likely to promote a normal phenotype in these cells. In CRC cell lines, butyrate enhances cellular differentiation and reduces proliferation [88,89]. In human studies, butyrate and the associated lowering of luminal pH are correlated with a reduced risk of CRC [90,91]. A specific strain [MDT-1] of the ruminal bacterium *Butyrivibrio fibrisolvens* has been evaluated for use as a probiotic to prevent CRC cancer since it produces high amounts of butyrate [92]. Using a mouse model of colon cancer, the administration of MDT-1 has reduced the number of ACF and the percentage of mice with an increased proportion of ACF. Furthermore, the human probiotic *Propionibacterium spp.* has been shown to kill CRC cells through apoptosis *in vitro* via its metabolites, the SCFA, acetate and propionate [93,94]. However, synbiotics would be more active than probiotics alone in increasing the production of SCFA and consequently protect against CRC onset [10,95]. A possible explanation is that the interaction of the immunomodulating properties of probiotic bacteria and butyrate, which is more produced via fermentation of prebiotics, results in an upregulation of apoptosis [10,11]. In addition to SCFA, probiotics are involved in the production of another group of fatty acids, termed conjugated linoleic acids [CLAs]. These are a group of isomers of linoleic acid that have been shown to exert numerous health benefits, including anti-inflammatory and anti-carcinogenic effects [96,97]. In rodent studies CLA has been shown to reduce the incidence of colonic tumors [98,99]. Using animal models, Ewaschuk *et al. *[100] demonstrated that the probiotic strains in the mixture VSL#3 are able to convert linoleic acid into CLA, inducing the upregulation of PPARγ, a reduction in colonic tumor cells viability, and the induction of apoptosis. These studies support a role for supplemental probiotics as a strategy for preventing CRC by fermentation of indigestible food, but further investigations are needed.

### Inhibition of tyrosine kinase signaling pathways

Signaling pathways are represented by a series of biochemical events whereby a cell communicates with the extracellular environment. Signaling pathways are activated by receptors or cytoplasmic proteins with tyrosine kinase activity and play a critical role in carcinogenesis [101]. *Saccharomyces boulardii [*Sb*]* is a safety probiotic agent used to prevent or treat a wide variety of human GI disorders [102,103]. It has been reported that Sb acts through modulation of the host signaling pathways that regulate the intestinal mucosal inflammatory response. In particular, Sb down-regulates MAPK signaling pathways [104,105] that are located downstream of many growth-factor receptors, including the epidermal growth factor receptor [EGFR]. The EGF receptor family consists of four members: ErbB1/EGFR/HER1, ErbB2/HER2/Neu, ErbB3/HER-3 and ErbB4/HER-4 that are important for cancer development [106]. Chen *et al. *[107] wanted to examine the effects of Sb on tumor development in *Apc^Min^* mice, an animal model used for quantitative and mechanistic studies of the induction of intestinal tumors [108]. Sb prevented cancer cell colony formation, reduced EGF-mediated cell proliferation, and increased apoptosis. Both *in vitro* and *in vivo* effects were consistent with inhibition of the EGFR and Akt pathways. Furthermore, a laboratory study by Ma *et al. *[109] demonstrated that the probiotic *Bacillus polyfermenticus* suppressed colon cancer cells growth *in vitro* and colon cancer tumor growth *in vivo*. *Bacillus polyfermenticus* exerted its anticancer effect through the reduction of ErbB2 and ErbB3 and their downstream signaling molecules E2F-1 and cyclin D1. Thus, in addition to the other anti-tumorigenic effects, probiotics may inhibit EGFR and other tyrosine kinase signaling pathways and thereby may also serve a novel therapeutic or prophylactic role in intestinal malignancies.

## Conclusions

Although a wide range of studies have brought to growing remarkable findings in recent years, it has not still been possible to obtain conclusive clinical evidence supporting the role of probiotics in CRC prophylaxis. Since CRC is an impractical endpoint in terms of numbers of subjects, cost, study duration and ethical considerations, probiotic intervention studies often use recurrence of preneoplastic lesions or intermediate biomarkers of cancer as an endpoint [110,111]. Several mechanisms could explain the preventive action of probiotics against CRC onset. All of the CRC-preventing mechanisms previously discussed are supported in varying degrees from *in vitro* and animal model studies, some of them even from human clinical studies. We are not still able to determine which mechanisms are most effective. Most likely distinct strains of probiotics operate with specific mechanisms. Further investigations are strongly required in order to establish the impact of each mechanism and the real usefulness of probiotics in CRC prevention.

## Competing interests

The authors declare that they have no competing interests.

## Authors' contributions

MU: conception and design, drafting the manuscript, given final approval of the version to be published; GM, VDA, MM, MV: drafting the manuscript, given final approval of the version to be published; FB, GB, FD, AB: critical revision, given final approval of the version to be published.
